# Targeted Infection Control Practices in Japanese Hospitals for Multidrug-Resistant Organisms: Guidance From the Public Health Center

**DOI:** 10.7759/cureus.50680

**Published:** 2023-12-17

**Authors:** Ayako Nakayama, Ichiro Yamaguchi, Koji Okamoto, Shigefumi Maesaki

**Affiliations:** 1 Department of Administration, Kawaguchi Public Health Center, Kawaguchi City, JPN; 2 Department of Environmental Health, National Institute of Public Health, Saitama, JPN; 3 Department of Infectious Disease and Infection Control, Saitama Medical University, Saitama, JPN

**Keywords:** training session, infection control practices, guidance, useful information, multidrug-resistant organisms, public health center

## Abstract

Introduction

The study conducted by the Kawaguchi City Public Health Center (PHC) in 2023 on hospital infection control (IC) programs revealed that hospitals can improve their IC programs if the PHC provides training sessions (TSs) that have numerical effects. In this study, we expected that we could help hospitals develop their IC practices by providing targeted guidance. This study aimed to clarify targeted guidance on IC practices and TS programs to develop hospitals'hospitals' IC programs on multidrug-resistant organisms (MDROs) by examining hospitals'hospitals' IC programs in reference to the study conducted in 2023 and other case reports.

Methods

In June 2022, the Kawaguchi City PHC conducted TSs for 19 hospitals and eight affiliated (AFs) clinics with beds, providing guidelines and practices on infection control (IC) for MDROs. After the TSs, we sent a questionnaire to these hospitals and affiliated clinics. The questionnaire inquired about current and planned IC policies, hand hygiene compliance programs (HHCPs), the usefulness of the TSs conducted by the PHC, and IC programs that the facilities intended to implement or develop in the future. This study examined the relationship between the perceived usefulness of the information provided and the IC programs planned for development, referencing a study conducted in 2023 and other case reports.

Results

Seventeen hospitals and six AFs with beds responded to the survey, yielding an 85.2% response rate. IC policies for methicillin-resistant Staphylococcus aureus (MRSA) were prepared by 21 hospitals (91.3%), whereas only five hospitals (21.7%) had prepared IC policies for carbapenem-resistant Enterobacteriaceae. Regarding HHCPs, an increase in the availability of alcohol-based hand sanitizer was identified by 17 hospitals (73.9%), while 13 hospitals (56.5%) reported using posters or symbols, 12 hospitals (52.2%) reported using TS and hand sanitizers, and nine hospitals (39.1%) assessed HH compliance and provided feedback. Furthermore, nine hospitals (39.1%) identified HHCPs and Environmental Cleaning (EC) for carbapenemase-producing Enterobacteriaceae (CPE) as useful information. There was a statistically significant association between TSs on HHCPs and the development of new HHCPs (p = 0.027). Additionally, information on EC for CPE was significantly associated with the development of staff cohorting strategies (p = 0.007). However, TS programs were not significantly connected to EC, nor were TSs to be developed.

Conclusion

The PHC should advise hospitals to assess if their HHCPs effectively contribute to improving HH compliance. It is essential for the PHC to furnish hospitals with resources and information that aid in the development of EC training. Additionally, the PHC should support the creation of specific and effective TS programs focused on EC or TS development. Conducting surveys to identify barriers to implementing staff cohorting strategies is also recommended. We propose that TS programs should include quantifiable data on HHCPs and EC, such as

## Introduction

In the United States, local public health departments are implementing infection control (IC) strategies in facilities with high rates of carbapenem-resistant Enterobacteriaceae (CRE) to prevent CRE infections [1]. In Japan, the central offices of local public health departments or public health centers (PHCs) routinely visit hospitals and affiliated clinics (AFs) with beds (hospitals) as mandated by the Japanese Medical Care Act. There were 32 hospitals in Kawaguchi city in June 2022. Routine visits to these hospitals by the Kawaguchi PHC revealed that most of them had not prepared written IC policies for multidrug-resistant organisms (MDROs) and had not developed hand hygiene compliance programs (HHCPs) [2]. Since 2020, however, approximately 2,000 cases of CRE have been notified to the Japanese public health authorities [3]. In addition, the identified CRE cases reported by 25% of Japan's hospitals numbered over 9,000 [2]. Therefore, the Kawaguchi PHC concluded that information about preventing the spread of CRE infection should be provided not only to the hospitals in which CRE has been identified but also to those where it has never been identified [2]. Kawaguchi, located near Tokyo, lacks university hospitals and only has a few infection preventionists [2]. Therefore, it can be challenging for the hospital personnel in Kawaguchi to access advice from IC specialists [2]. Local public health authorities have not reported whether the information provided by them on IC had any effect on IC programs implemented by the hospitals, so to address the lack of information in Kawaguchi, we decided to examine whether training session (TS) programs provided by the PHC have had any effect on the development of IC programs created by the hospitals [2]. In June 2022, the Kawaguchi PHC provided hospitals with TSs on IC guidelines and practices, focusing specifically on MDROs, followed by a survey of the IC programs by the hospitals [4]. We mentioned the results of this study, which was presented at the Nihon Kousyuueisei Gakkai Annual Meeting in 2022, in a related study conducted in 2023 [2]. However, in the 2022 study, we did not specifically examine the impact of effective TS programs on the development of IC programs in hospitals. This situation led us to conclude that we needed to examine whether the study results conducted in 2023 were compatible with the 2022 study results to determine the generalizability of the study. Meanwhile, in the 2023 study, we planned to develop the hospital's IC programs by providing effective TS programs by the PHC [2]. In this study, our objective is to assist hospitals in developing their IC practices by providing targeted guidance. Additionally, we anticipated that the guidance from the PHC on the hospitals' IC practices could be more effectively targeted by analyzing the results of the 2022 study in relation to those of the 2023 study. The primary aim of the current study is to elucidate the targeted guidance programs on IC practices and TS programs. These programs are intended to enhance IC program development by examining HHCPs and IC programs in the context of the 2023 study [2], as well as other case reports. This article was previously presented as a meeting poster at the 2022 Nihon Kousyuueisei Gakkai Annual Meeting on October 8, 2022.

## Materials and methods

In June 2022, the Kawaguchi PHC conducted TS programs on IC practices for MDROs for 19 hospitals and eight AFs with beds in Kawaguchi via a web conference system facilitated by a public health physician (Table 1). The TS participants were medical doctors, pharmacists, nurses, and hospital clinical laboratory technicians. After the TS programs, we emailed a questionnaire to 19 hospitals and eight AFs with beds. In the email, we explained that the respondents' identifying information would be removed from the questionnaire results before they were made public, and we asked for their consent to participate in our study. Seventeen hospitals and six AFs with beds responded to the survey. The hospitals' responses to the questionnaire covered the following topics: preparedness for the IC written policies on MDROs and for policies the hospitals intended to prepare, HHCPs by the hospitals, IC programs that the hospitals intended to implement in the future or develop (to be developed), and useful information for hospitals that are developing IC programs (useful information).

**Table 1:** TS Programs Focused on IC for CRE and CPE by Kawaguchi PHC. TS: Training Session; IC: Infection Control; CRE: Carbapenem-resistant Enterobacteriaceae; CPE: Carbapenemase-producing Enterobacteriaceae; PHC: Public Health Center; MRSA: Methicillin-resistant Staphylococcus aureus; CP: Contact Precautions; MDRA: Multidrug-resistant Acinetobacter; MDROs: Multidrug-resistant Organisms; EC: Environmental Cleaning; PCR: Polymerase Chain Reaction.

Description	TS programs
Hand hygiene compliance programs	The hospitals need to implement feasible goal-setting that is accepted by the staff: an increase of about 20% in the hand hygiene compliance rate is recommended.
IC policy on MRSA by the university hospitals including CP	Specific criteria for private room management for MRSA patients; Specific criteria for dedicated equipment for MRSA patients; Minimize patient care items and equipment in MRSA patients’ rooms; Minimize the movement of patient care items and equipment from MDRO patients' rooms
IC on MDROs	If MDROs, including CRE, are identified, the hospital must target IC to prevent outbreaks.
IC and practice for CRE identified (one case)	Management of private room, informing the PHC, examining if the patient was infected after admission to the hospital, evaluating currently implemented IC, assessing whether transmission is occurring, identifying CRE contacts.
The definition of contact of CPE	Roommates, those who have shared the same toilet, those who have shared the same ward, etc.
Isolation for CRE or CPE	If CPE is identified, isolate the patients.
	If CPE is not identified, hospitals should consider or carry out isolation, considering the potential for outbreak involvement, evaluation of CP, and plasmid carrying the CPE gene.
EC for CPE	CPE infection was associated with previous CPE identification in the toilet of the patient’s room. Enhanced EC of the toilet is recommended.
	Use disposable or dedicated patient care equipment.
IC and CPE practice identified (more than two cases)	Hospitals should identify common factors between CPE cases, block the source of infection, and consider or perform environmental screening.
The clearance of CPE carriage	Patients with no risk factors who are readmitted more than 12 months after a positive CPE colonization result are required to have three negative screening swabs taken at least 24 hours apart.
	Patients are required to provide negative results on two rectal swabs submitted for culture and one swab submitted for PCR.
Enhanced IC programs	Hospitals need to develop IC programs as follows:
	1. Prepare a written IC policy on CRE in reference to international guidelines and disseminate it to all employees via their TS.
	2. Develop IC practices for CRE and CPE and disseminate them to all employees via the TSs.

Variables excluding the IC written policy were all summarized using the number of hospitals. The IC policy was summarized for each MDRO individually. Multiple and single regression analyses were used to determine the relationship between IC programs to be developed and useful information. Furthermore, a correlation analysis was carried out to determine the relationships between IC programs to be developed and useful information. We used BellCurve for Excel (BellCurve, Tokyo) for multiple and single regression analyses and correlation analysis. We performed a Spearman's rank correlation coefficient for the correlation analysis. We examined the HHCPs and IC programs implemented by hospitals, referencing a related study conducted in 2023 and various other case reports. The PHC in Japan plays a crucial role in ensuring hospital safety by guiding them to develop IC programs and practices [2]. In this study, our goal was to provide effective guidance on IC practices and TS to help hospitals within our jurisdiction develop their IC programs. We analyzed the survey results, which were categorized as city activities in Kawaguchi under the Medical Care Act, and evaluated them against recommendations from research reports and established best practices. This study involved no invasive procedures, such as drawing blood, collecting samples, or asking traumatic questions. As we did not use human subjects in our research, approval from the ethics committee was not required. 

## Results

**Figure 1:**
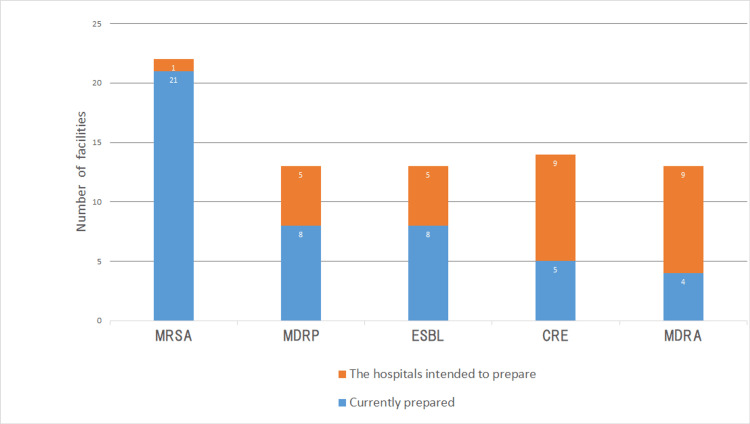
Infection control policies on MDROs (n=23) MDRO: Multidrug-resistant organisms; MRSA: Methicillin-resistant *Staphylococcus aureus*; MDRP: Multiple drug-resistant *Pseudomonas aeruginosa*; ESBL: Extended spectrum beta-lactamases; CRE: Carbapenem-resistant Enterobacteriaceae; MDRA: Multidrug-resistant *Acinetobacter*. Data are presented as number of facilities.

Twenty-three hospitals responded to the survey for a response rate of 85.2%. IC policies for MRSA were prepared by 21 hospitals (91.3%), whereas five hospitals (21.7%) had prepared the IC policy on CRE and four hospitals (17.4%) for MDRA (Figure 1). Nine hospitals (39.1%) intended to prepare for the IC policy on CRE, and nine hospitals (39.1%) for MDRA (Figure 1).

**Figure 2:**
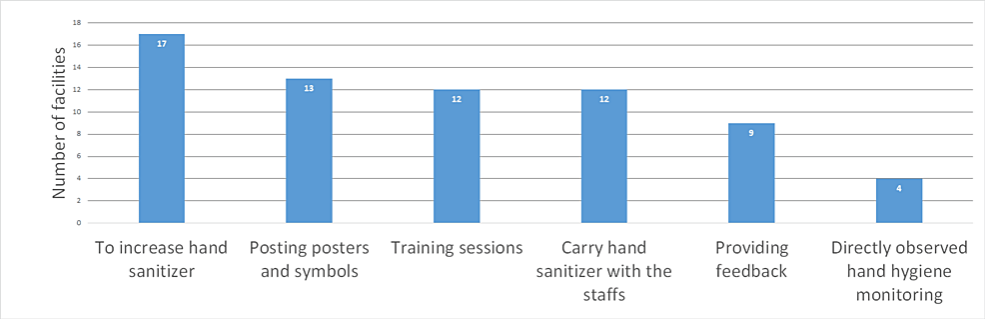
Hand hygiene compliance programs (n=23). To increase hand sanitizer; to increase the opportunity to utilize hand sanitizer. Data are presented as number of facilities.

**Figure 3:**
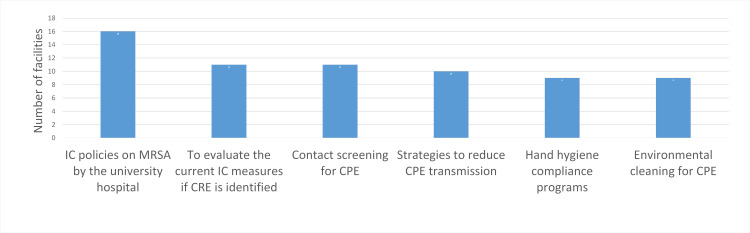
Useful information for developing IC programs by the hospitals (n = 23). IC: Infection control; MRSA: Methicillin-resistant *Staphylococcus aureus*; CRE: Carbapenem-resistant Enterobacteriaceae; CPE: Carbapenemase-producing Enterobacteriaceae. Data are presented as number of facilities.

**Figure 4:**
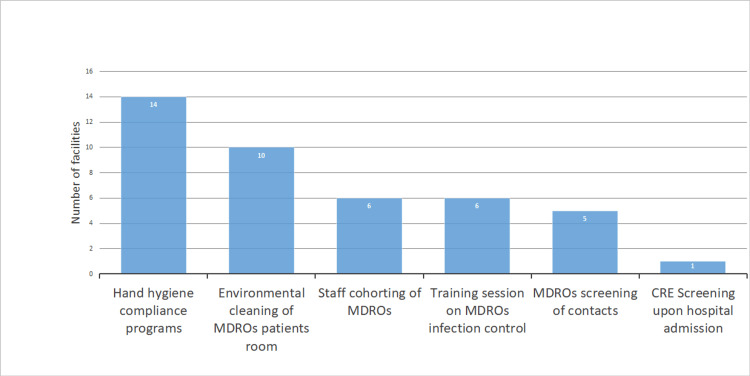
Infection control programs that hospitals intend to implement or develop in the future. MDROs: Multidrug-resistant organisms; CRE: Carbapenem-resistant Enterobacteriaceae. Data are presented as number of facilities.

The HHCPs implemented by the hospitals are summarized in Figure 2. Seventeen hospitals (73.9%) reported an increase in the availability of alcohol-based hand sanitizer, making it the most frequently implemented program. Posters and symbols promoting HH were used by 13 hospitals (56.5%), TSs by 12 hospitals (52.2%), hand sanitizer carried by staff by 12 hospitals (52.2%), and an assessment of HH compliance with feedback provided to staff by nine hospitals (39.1%). Figure 3 illustrates valuable insights for developing hospital IC programs; 16 hospitals (69.6%) found IC policies on MRSA, including contact precautions stipulated by university hospitals, to be useful. Additionally, EC for CPE was considered useful by nine hospitals (39.1%), as were HHCPs (39.1%; Figure 3). The IC programs identified for future development include HHCPs by 14 hospitals (60.9%), EC of patient rooms by 10 hospitals (43.5%), staff cohorting for MDROs by six hospitals (26.1%), and TSs on MDROs IC by six hospitals (26.1%; Figure 4).

Table 2 shows the relationships between useful information and the IC programs to be developed. The association between useful information, such as HHCPs, and the development of HHCPs was statistically significant, with a regression coefficient (RC) of 0.46, a p-value of 0.027, and a 95% CI of 0.06-0.86. However, useful information like IC policies on MRSA did not significantly correlate with the development of IC programs such as HHCPs, EC of patients' rooms, staff cohorting, and TSs. Furthermore, the useful information 'EC for CPE' was significantly associated with staff cohorting (standard partial RC = 0.55, p = 0.007, 95% CI = 0.17-0.93). There exists a moderate correlation among useful information like HHCPs, IC policies on MRSA, and EC for CPE, with correlation coefficients of 0.337, 0.270, and 0.337, respectively (Table 3). A strong correlation was found between the EC of the rooms of MDRO patients and staff cohorting, with a correlation coefficient of 0.677 (Table 4).

**Table 2:** Association between useful information for developing IC programs and IC programs that the hospitals intended to implement or develop in the future. IC: Infection control; RC: Regression coefficient; CI: Confidence interval; HHCPs: Hand hygiene compliance programs; MRSA: Methicillin-resistant *Staphylococcus aureus*; CP: Contact precaution; EC: Environmental cleaning; CPE: Carbapenemase-producing *Enterobacteriaceae*; MDROs: Multidrug-resistant organisms. Statistical significance determined as p≤ 0.05.

Dependent variables	Independent variables	Multiple regression analysis				Single regression analysis			
IC programs that the hospitals intended to implement in the future or develop	Useful information for developing IC programs by the hospitals	Standard partial RC	t value	P-value	95% CI	RC	t value	P-value	95% CI
HHCPs	HHCPs	0.4	1.85	0.079	-0.05-0.85	0.46	2.38	0.027	0.06-0.86
	IC policies on MRSA by university hospitals, including CP	0.06	0.27	0.792	-0.43-0.55	0.26	1.15	0.262	-0.21-0.73
	EC for CPE	0.15	0.7	0.495	-0.30-0.60	0.28	1.33	0.199	-0.16-0.71
EC of the MDROs patients’ room	HHCPs	-0.24	-0.99	0.335	-0.74-0.26	-0.17	-0.76	0.454	-0.62-0.29
	IC policies on MRSA by university hospitals, including CP	0	0	1	-0.54-0.54	0.01	0.04	0.97	-0.48-0.50
	EC for CPE	0.26	1.09	0.288	-0.24-0.76	0.2	0.91	0.372	-0.25-0.65
Staff cohorting for MDROs	HHCPs	0.05	0.28	0.784	-0.33-0.43	0.12	0.61	0.547	-0.29-0.52
	IC policies on MRSA by university hospitals, including CP	-0.25	-1.28	0.216	-0.66-0.16	-0.04	-0.17	0.865	-0.47-0.40
	EC for CPE	0.55	3.05	0.007	0.17-0.93	0.48	2.93	0.008	0.14-0.83
Training sessions on MDROs	HHCPs	0.05	0.24	0.815	-0.39-0.49	-0.06	-0.32	0.749	-0.47-0.34
	IC policies on MRSA by university hospitals, including CP	-0.19	-0.30-0.60	0.422	-0.67-0.29	-0.24	-1.2	0.245	-0.66-0.18
	EC for CPE	-0.2	-0.95	0.354	-0.64-0.24	-0.25	-1.3	0.207	-0.64-0.15

**Table 3:** Correlation between useful information for developing IC programs by the hospitals. IC: Infection control; HHCPs: Hand hygiene compliance programs; MRSA: Methicillin-resistant *Staphylococcus aureus*; CP: Contact precautions; EC: Environmental cleaning; CPE: Carbapenemase-producing Enterobacteriaceae.

Variable 1	Variable 2	Spearman’s rank correlation coefficient (between variable 1 and 2)
HHCPs	IC policies on MRSA including CP	0.337
HHCPs	EC for CPE	0.270
IC policies on MRSA including CP	EC for CPE	0.337

**Table 4:** Correlation between IC programs that the hospitals intended to implement or develop in the future. IC: Infection control; HHCPs: Hand hygiene compliance programs; EC: Environmental cleaning; MDROs: Multidrug-resistant organisms.

Variable 1	Variable 2	Spearman’s rank correlation coefficient (between variable 1 and 2)
HHCPs	EC of the MDROs-identified patients’ room	-0.195
HHCPs	Staff cohorting for MDROs	0.071
HHCPs	Training sessions on MDROs	-0.335
EC of the MDROs patients’ room	Staff cohorting for MDROs	0.677
EC of the MDROs patients’ room	Training sessions on MDROs	0.078
Staff cohorting for MDROs	Training sessions on MDROs	0.098

## Discussion

The importance of hospitals preparing a written IC policy on MDROs

In Japan, there are no national guidelines for IC on MDROs. Moreover, the WHO does not recommend that hospitals prepare written IC policies specifically for MDROs [5]. Meanwhile, the EU, Australia, and some states in the United States have developed guidelines on MDROs [6-8]. Therefore, hospitals in these regions can prevent and control MDROs with reference to these guidelines. We suggest that hospitals in Kawaguchi, apart from the five hospitals (21.7%) that have prepared IC policies for CRE (Figure 1), may not be adequately equipped to prevent and control CRE. In Japan, 12.2% of hospitalized patients have been colonized with CRE [9]. Considering this, hospitals in Kawaguchi, where CRE has never been identified, might encounter CRE cases in the future. We recommend that the Kawaguchi PHC continues to encourage hospitals to develop CRE-specific IC policies. Additionally, public health authorities, including the PHC, and IC specialists, who advise hospitals on IC, should recommend that hospitals prepare written IC policies on MDROs, especially if they are not covered by national guidelines. In this study, nine (39.1%) hospitals intended to prepare IC policies for CRE (Figure 1). Training in IC was favorably correlated with greater knowledge of MDROs and IC among healthcare workers [10]. Therefore, we propose that participation in the TSs carried out in this study may contribute to preparing IC policies on CRE.

Promotion of HH compliance programs by the hospitals

In Japan, there are no national guidelines for HH compliance. The most essential factors in the development of HH compliance are the location of the hand sanitizer close to the room entrance and its easy visibility [11]. Therefore, if the hospitals increase the opportunities to use hand sanitizer (Figure 2) but do not place it at the room entrances or make it easily visible, HH compliance might not improve as expected. The WHO does not require staff to carry hand sanitizer and recommends that hospitals adapt resources that are effective for tailoring HH compliance for the local context [12]. In hospitals in Japan, the staff's policy of carrying hand sanitizer (Figure 2) increased hand sanitizer usage [13]. After examining its effectiveness for developing HH compliance, public health authorities and IC specialists need to recommend that hospitals introduce portable hand sanitizers. The importance of posters about HH is emphasized by the WHO [12]. However, there is no recommendation about the contents of posters encouraging HH compliance [12]. The introduction of the Apple emoji, which is the symbol that indicates the timing of HH for hospital personnel, contributed to improved HH compliance [14]. Hospitals in Kawaguchi that post posters or symbols (Figure 2) should examine their impact on HH compliance. Public health authorities and IC specialists need to advise hospitals on the effective placement of posters or symbols related to HH compliance and must guide hospitals in examining the effect of posters and symbols. The effect of feedback on HH compliance is emphasized by the WHO [12]. However, there is no recommendation on the specific content of feedback related to developing HH compliance [12]. Specific feedback programs useful for developing HH compliance have been reported [15], [16], [17]. Therefore, public health authorities and IC specialists should recommend that hospitals examine whether their provision of feedback, as currently conducted (Figure 2), could contribute to the development of HH compliance. The TS program, "the prevention of healthcare-associated infections (HAI) and cost savings by implementing cleaning bundles," resulted in a strong association with HHCPs to be developed by the hospitals [2]. The financial impact of the enhanced IC practices within the organization encourages the hospital staff to prevent the spread of MDROs [18]. Therefore, we suggest that TS programs in this study, resulting in about a 20% HH compliance rate, should be increased. However, they are not significantly associated with developing HHCPs by multiple regression analysis (Table 2), so they might not be effective in developing HHCPs, given the lack of information on cost-effectiveness. The public health authorities and IC specialists need to provide hospitals with information on the cost-effectiveness of HHCPs and should guide hospitals to provide their staff with such information.

Promotion of EC by the hospitals

The inadequately written EC policies developed by hospitals with insufficient knowledge of EC, and EC practices by workers at co-signed businesses (CBs) might be barriers to improving EC in hospitals in Japan [2]. Therefore, we suggest that these findings might have contributed to the lack of Training Sessions (TS) programs related to EC developed by the Kawaguchi Public Health Council (PHC), as observed in the hospitals (Table 2). In Japan, there are no national guidelines for EC of Multidrug-Resistant Organisms (MDROs). However, the World Health Organization (WHO) has stated that training cleaning staff is crucial for improving EC in hospitals [5]. Regarding Infection Control (IC) for Carbapenemase-producing Enterobacteriaceae (CPE) outbreaks, multi-component IC measures, including enhanced EC, have been used, and their effectiveness has been established [19]. Hospitals can base their TS programs on their IC policies for EC. We suggest that hospitals develop their IC policies for EC by referencing EC measures implemented during MDRO outbreaks. Public health authorities and IC specialists must provide hospitals with information not only on EC in toilets (Table 1) but also on EC measures conducted during outbreaks to support the development of EC training in hospitals.

Promotion of staff cohorting by the hospitals

The WHO does not mention the importance of staff cohorting for the prevention and control of MDROs [5]. Meanwhile, IC practices that included staff cohorting were effective in controlling an outbreak of CPE [20]. We could not find previous studies mentioning the barriers to implementing staff cohorting. We suggest that a low incidence of CRE might have resulted in only six hospitals intending to implement or develop staff cohorting (Figure 4). However, this study revealed that TS programs such as EC for CPE (the recommendation for enhanced EC of toilets) might encourage the implementation of staff cohorting by the hospitals (Table 2). We suggest that the hospitals had not expected that the toilet could be the source of infection, and the TS programs on EC for CPE had prompted them to prepare for an MDRO outbreak plan by practicing staff cohorting. The public health authorities and IC specialists should conduct a survey on the barriers to implementing staff cohorting and provide the hospitals with the survey results.

Promotion of TSs on MDROs by the hospitals

In Japan, there are no national guidelines for TSs on MDROs. However, staff training is critical for the implementation of successful MDROs control, as mentioned in the WHO guidelines [5]. The lack of specific and effective TS programs by the PHC might have contributed to TS programs by the PHC that were not related to the TSs that the hospitals intended to conduct [2]. In this study, specific effective TS programs by the PHC were not provided. The public health authorities and IC specialists must examine the effectiveness of their specific TS programs by asking why the hospitals develop TSs and why they do not so they may provide effective information on the TSs on MDROs.

Targeted guidance to promote the development of IC programs

We examined the HHCPs and IC programs offered by the hospitals in reference to a related study conducted in 2023 [2] and other case reports. The related study conducted in 2023 revealed that the TS programs provided by the PHC developed IC programs such as HHCPs and HH compliance feedback but did not result in the development of EC and TSs by the hospitals [2]. Moreover, the authors of the related study conducted in 2023 examined the reasons why the TS programs provided by the PHC were related or not related to the IC programs to be developed and found three factors: HHCPs developed based on the effect of IC practices, barriers such as the lack of knowledge about EC, and TSs to be developed that were impeded by the lack of specific and effective TS programs [2]. The findings and case reports cited in this study provided us with several insights that enabled us to discuss the targeted guidance on IC practices by the hospitals; these include guiding hospitals to examine the effect of HHCPs, guiding hospitals to provide their staff with cost-effective HHCPs, providing hospitals with information about improving EC training, conducting a survey on the barriers to implementing staff cohorting. We suggest that the guidance programs discussed in this study could help hospitals develop IC practices using a novel method: guiding hospitals to examine the effect of HHCPs and provide their staff with cost-effective HHCPs and providing the hospitals with information for improving EC training and conducting a survey on barriers to staff cohorting. These results provided an additional point of view regarding developing IC programs on which a related article discussed the effectiveness of TS programs [2].

Providing quantified information to promote the development of IC programs

In this study, the TS programs (including HHCP, aiming for a HH compliance rate increase of about 20%, and EC for CPE, which recommends enhanced EC for toilet-associated CPE infections identified previously) were associated with the development of HHCPs and staff cohorting (Table 2). These TS programs also featured IC practices or epidemiological findings, providing quantified information on HHCPs and evidence upon which EC enhancement should be based. IC programs under development should be linked to the provision of information about numerical effects: specific feedback that is effective for HHCPs, preventing Healthcare-Associated Infections (HAI), allowing cost savings through cleaning bundles implementation, and assessing the impact of TS programs on IC, such as reductions in MDROs [2]. Therefore, we suggest that providing quantified information on IC practices or findings might contribute to the development of IC programs targeting MDROs in hospitals. Public health authorities and IC specialists should provide hospitals with TS programs, including IC practices or findings on IC with quantified information, to assist in the development of their IC programs. The organizational indicator for IC practices and programs developed by hospitals is that IC programs should undergo review [21]. Therefore, we need to conduct a survey on preparedness for IC policies on MDROs (Figure 1), IC policies on EC, HHCPs (Figure 2), and TSs, to examine the effect of guidance and TS programs.

Japanese national policy on IC and the importance of this study

The Japanese Ministry of Health, Labor, and Welfare has established a policy stating that IC specialists, who primarily study in universities, should address issues with IC practices in hospitals in coordination with public health physicians [2]. However, in areas of Japan like Kawaguchi, where there are no university hospitals, coordinating with IC specialists from universities outside the local jurisdiction and receiving advice on solving local issues can be challenging. Therefore, providing targeted guidance on IC practices and effective TS programs for IC programs in this study could improve the IC practices and programs in jurisdictions where advice from university-based IC specialists is not available. In the United States, local public health authorities are responsible for educating hospitals about MDROs [22]. Our study did not find TS programs by local public health authorities addressing issues with IC programs [2]. We suggest that some districts may face challenges similar to those revealed by this study with IC practices and programs. Therefore, targeted guidance on IC practices and TS programs focusing on IC programs that address MDROs might enable local public health authorities to provide more effective information on IC practices and programs.

Strengths and limitations of this study

In this study, we found that hospitals could develop their own IC programs when the PHC provided information on HHCPs and EC for CPE. Moreover, we indicated that the public health authorities and IC specialists must guide the hospitals to examine the effectiveness of the HHCPs that are currently being conducted. Regarding the development of staff cohorting, we proposed that the public health authorities and IC specialists conduct a survey on the barriers to implementing staff cohorting and provide the hospitals with the survey results. Moreover, we suggested that the public health authorities and IC specialists should provide the hospitals with information on EC measures conducted during the outbreak to support the hospitals' development of EC training. Regarding TSs by the hospitals, we suggested that public health authorities and IC specialists must examine the effectiveness of their specific training programs by asking why the hospitals develop TSs and why they do not. Furthermore, we found that public health authorities and IC specialists must provide quantified information on HHCPs and evidence, based on which the EC should be enhanced to address issues in IC programs. The survey response rate in this study was relatively high at 85.1%, enabling us to collect comprehensive information on IC programs from hospitals throughout the jurisdiction. We expect that the targeted guidance program and the specific, effective information on IC programs discussed in this study will assist hospitals in other districts in developing their IC practices and programs. This study has several limitations. First, the data collected represent only 23 hospitals in Kawaguchi and may not fully represent IC practices and programs in other regions. Second, the PHC's policy of providing guidance and information to hospitals' IC practices and programs was restricted to a specific set of hospitals. Third, TS programs not related to EC might not be applicable in other districts where the CB does not implement EC [2]. Therefore, TS programs on EC should be examined in these districts to determine their applicability and effectiveness. Fourth, we did not examine preparedness for IC policies on MDROs or on EC, HHCPs, and TSs that are currently being conducted to determine the effect of guidance and TS programs. Therefore, we must acknowledge that the findings and TS programs implemented in this study might not directly apply to other jurisdictions or hospitals. However, we expect that the program implemented in this study, which aimed to provide the hospitals with targeted guidance on IC practices and effective TS programs for developing IC programs, will serve as a model that other jurisdictions could consider and adopt. Additional surveys and evaluations in different settings will help determine the generalizability and effectiveness of the strategies used in this study.

## Conclusions

This study should be examined in other districts where CBs do not implement EC, and its effects should be assessed. However, we can conclude that the targeted guidance on IC practices and TS programs, such as HHCPs and EC for CPE, could support hospitals in developing their IC practices and programs. In light of our findings, we suggest that the PHC continue to provide hospitals under its jurisdiction with targeted guidance on IC practices and TSs, including quantified information that is effective for helping hospitals enhance their IC programs.

## References

[1] (2023). Hospital-acquired infections - New York State. https://www.health.ny.gov/statistics/facilities/hospital/hospital_acquired_infections/.

[2] Nakayama A, Yamaguchi I, Okamoto K, Maesaki S (2023). Public health centers’ training session programs to develop programs on infection control practices for multidrug-resistant organisms in hospitals in Kawaguchi city, Japan. Cureus.

[3] (2023). The national epidemiological surveillance of infectious diseases. Category V required reporting infectious diseases. https://www.niid.go.jp/niid/ja/ydata/11530-report-ja2021-30.html.

[4] (2023). Release: survey results regarding training sessions on infection control issues discovered by annual inspection required by the medical service act (Iryo-hou). https://www.city.kawaguchi.lg.jp/soshiki/01090/018/iji/oshirase/41987.html.

[5] (2023). Guidelines for the prevention and control of carbapenem-resistant Enterobacteriaceae, Acinetobacter baumannii and Pseudomonas aeruginosa in health care facilities. http://https://www.who.int/publications/i/item/9789241550178.

[6] Magiorakos AP, Burns K, Rodríguez Baño J (2017). Infection prevention and control measures and tools for the prevention of entry of carbapenem-resistant Enterobacteriaceae into healthcare settings: guidance from the European Centre for Disease Prevention and Control. Antimicrob Resist Infect Control.

[7] (2023). Recommendations for the control of carbapenemase-producing enterobacterales (2021 CPE Guide). https://www.safetyandquality.gov.au/sites/default/files/202206/recommendations_for_the_control_of_carbapenemaseproducing_enterobacterales_final_accessible_pdf_version_november_20212.pdf.

[8] (2023). Carbapenem-resistant enterobacteriaceae (CRE) and other carbapenem-resistant organisms. CRE guideline (PDF). https://doh.wa.gov/sites/default/files/2022-02/420-097-Guideline-CRE.pdf.

[9] Yamamoto N, Asada R, Kawahara R (2017). Prevalence of, and risk factors for, carriage of carbapenem-resistant Enterobacteriaceae among hospitalized patients in Japan. J Hosp Infect.

[10] Vaillant L, Birgand G, Esposito-Farese M (2019). Awareness among French healthcare workers of the transmission of multidrug resistant organisms: a large cross-sectional survey. Antimicrob Resist Infect Control.

[11] Cure L, Van Enk R (2015). Effect of hand sanitizer location on hand hygiene compliance. Am J Infect Control.

[12] (2023). WHO guidelines on hand hygiene in health care. https://iris.who.int/bitstream/handle/10665/44102/9789241597906_eng.pdf?sequence=1.

[13] Shimada I, Banno M, Kumoi N (2009). Examination of hygiene procedures and hand roughness in hand-hygiene using portable hand-rub gel. Nihon Kankyoeisei Gakkaishi.

[14] Lotfinejad N, Tartari E, Sauser J, Fankhauser-Rodriguez C, Pires D, Pittet D (2022). Are emojis ready to promote the WHO 5 moments for hand hygiene in healthcare?. Antimicrob Resist Infect Control.

[15] Diefenbacher S, Fliss PM, Tatzel J, Wenk J, Keller J (2019). A quasi-randomized controlled before-after study using performance feedback and goal setting as elements of hand hygiene promotion. J Hosp Infect.

[16] Pada SM, Chee PL, Rathenam S, Ng KS, Alenton LS, Poh L, Tambyah PA (2019). Effectiveness of a ward level target accountability strategy for hand hygiene. Antimicrob Resist Infect Control.

[17] Fish L, Bopp D, Gregory D, Kerley KD, Gakhar S, Lavigne MC, Boyd F (2021). Hand hygiene feedback impacts compliance. Am J Infect Control.

[18] Lyles RD, Moore NM, Weiner SB (2014). Understanding staff perceptions about Klebsiella pneumoniae carbapenemase-producing Enterobacteriaceae control efforts in Chicago long-term acute care hospitals. Infect Control Hosp Epidemiol.

[19] French CE, Coope C, Conway L (2017). Control of carbapenemase-producing Enterobacteriaceae outbreaks in acute settings: an evidence review. J Hosp Infect.

[20] Tzouvelekis LS, Markogiannakis A, Psichogiou M, Tassios PT, Daikos GL (2012). Carbapenemases in Klebsiella pneumoniae and other Enterobacteriaceae: an evolving crisis of global dimensions. Clin Microbiol Rev.

[21] Zingg W, Holmes A, Dettenkofer M (2015). Hospital organisation, management, and structure for prevention of health-care-associated infection: a systematic review and expert consensus. Lancet Infect Dis.

[22] (2023). Interim Local Health Department (LHD) HAI/AR Strategy. https://www.cdc.gov/hai/hai-ar-programs/resources/local-strategy/index.html.

